# Alzheimer's Disease and HLA-A2: Linking Neurodegenerative to Immune Processes through an In Silico Approach

**DOI:** 10.1155/2014/791238

**Published:** 2014-08-17

**Authors:** Ricardo A. Cifuentes, Juan Murillo-Rojas

**Affiliations:** Area of Basic Sciences, Faculty of Medicine, Universidad Militar Nueva Granada, Bogotá, Colombia

## Abstract

There is a controversial relationship between HLA-A2 and Alzheimer's disease (AD). It has been suggested a modifier effect on the risk that depends on genetic loadings. Thus, the aims of this study were to evaluate this relationship and to reveal genes associated with both concepts the HLA-A gene and AD. Consequently, we did first a classical systematic review and a meta-analysis of case-control studies. Next, by means of an in silico approach, we used experimental knowledge of protein-protein interactions to evaluate the top ranked genes shared by both concepts, previously found through text mining. The meta-analysis did not show a significant pooled OR (1.11, 95% CI: 0.98 to 1.24 in Caucasians), in spite of the fact that four of the included studies had a significant OR > 1 and none of them a significant OR < 1. In contrast, the in silico approach retrieved nonrandomly shared genes by both concepts (*P* = 0.02), which additionally encode truly interacting proteins. The network of proteins encoded by *APP, ICAM-1, ITGB2, ITGAL, SELP, SELL, IL2, IL1B, CD4*, and *CD8A* linked immune to neurodegenerative processes and highlighted the potential roles in AD pathogenesis of endothelial regulation, infectious diseases, specific antigen presentation, and HLA-A2 in maintaining synapses.

## 1. Introduction

Alzheimer's disease (AD) is a neurodegenerative process of the central nervous system (CNS) that is clinically characterized by an impairment of memory and other cognitive functions [1]. It is recognized as a multifactorial illness with both genetic and nongenetic causes. There have been identified four major genes associated with inherited risk for AD:* presenilin-1*,* presenilin-2*,* amyloid precursor protein *(*APP*), and* apolipoprotein E*. Mutations in these genes cause dysregulation of amyloid precursor protein processing, and in particular of the handling of a proteolytic derivative termed* beta-amyloid* (Abeta) that ultimately causes neuronal dysfunction and death [2].

Some findings also suggest an immune involvement in AD. Telomere length of T cells has been inversely correlated with cognitive performance impairment, apoptosis, serum levels of TNF-*α*, and the proportion of CD8^+^ T cells lacking expression of the CD28 costimulatory molecule [3]. There are augmented levels of CD8^+^ T memory cells, down regulation of CD8 receptors, and increased reactivity of CD4^+^ and CD8^+^ T-cells [4]. With regard to disease stages, there have been reported alterations in subsets of CD4^+^ cells in patients with mild AD, with decreased percentages of naive cells, elevated memory cells, and increased proportions of CD4^+^ cells lacking CD28. T potentially regulatory cells, CD4^+^CD25^+high^, with a naive phenotype are also reduced in AD patients [5]. It has been observed in patients of severe stage that there is a significant TNF-*α* increase in serum as well as a significant decrease in CD4^+^ lymphocytes [6].

Epidemiological data suggest that some determinants of AD might reside in genes from the human leukocyte antigen (HLA) that regulate immune inflammatory responses [7]. It has been described the association of AD with both HLA-B7 and HLA-A2 [1]. Some authors have also found increased frequency of HLA-A∗01 and HLA-DRB1∗03 alleles and decreased frequency of HLA-DRB1∗09 in late-onset AD cases [8, 9]. But these associations have shown no consistency among different ethnic groups [1]; nearly every positive result has been followed by several studies that have failed to replicate it or that have contradicted it [7]. In the case of HLA-DRB1∗03 and its linked TNF-*α* 2-1-2 haplotype (-308/A, -238/G and TNF-a2 polymorphisms), it has been described a protective effect against AD [10], contrary to the effect of the HLA-DRB1∗03 allele described above. Even more, some researchers have indicated that there is no compelling evidence of a strong, direct association between AD and any HLA class I or II allele [11]. Consequently, it has been suggested that there is a modifier effect on the risk that depends on genetic loadings and further analysis, considering both HLA and non-HLA genes, are therefore necessary [7, 10].

However, there is accumulated evidence that suggests the involvement of the* HLA-A* gene in the pathogenesis of AD. A meta-analysis of all studies available until the 2000th year supported previous evidence of an excess of HLA-A2 in AD [12]. More recently, it has been observed that HLA-A2 and APOE4 independently reduced the age at onset of AD through an effect that seems to be additive in a population from China [13] and that A2 homozygotes had an earlier onset of AD in a population from North-America [14]. With this panorama, the aims of this study were to evaluate the current evidence of the association between HLA-A2 and AD and reveal genes that can influence the relationship between HLA-A and AD, thus assisting to point out pathogenic pathways related to AD. Our analysis was made by means of a meta-analysis of case-control studies that evaluated this association, and by using experimental knowledge of protein-protein interactions to evaluate the top ranked genes that were shared by the concepts HLA-A and AD, which had previously been found through a text mining approach of the biomedical literature.

## 2. Materials and Methods

### 2.1. Search Strategy and Selection Criteria

A systematic review of electronic databases (PubMed, EMBASE) was done independently by two researchers. The final date for inclusion was June, 2013. The search strategy used MeSH terms and text words: “Alzheimer disease,” “Alzheimer's disease,” “Alzheimer,” and “HLA.” No other criteria were taken into account. The inclusion criteria were the following: (1) AD diagnosis established by using the* National Institute of Neurological and Communicative Disorders and Stroke* and* the Alzheimer's Disease and Related Disorders Association* (NINCDS-ADRDA), The* Consortium to Establish a Registry for Alzheimer's Disease* (CERAD) or the* Diagnostic and Statistical Manual* (DSM) criteria; (2) If AD diagnosis criteria mentioned in numeral 1 were not used, the article must mention that there was histopathological confirmation or that other causes of dementia were clinically excluded in the patients from the AD cohort; (3) indication in the title or in the abstract that a relationship between HLA and AD was evaluated; (4) case-control study design; (5) publication of sufficient original data on the HLA-A2 prevalence in cases and in controls to calculate reliable odds ratios (OR) [15]; (6) etiology of cases not related to the four major genes described in AD [2]; and (7) manuscript's publication in a peer-reviewed journal as a full paper.

### 2.2. Data Extraction and Meta-Analysis

The data collected from each study were as follows: first author and the year of study, country, the number of cases and controls typified as HLA-A2 or with the alternative classification of HLA-A. Calculations were done for each ethnic origin by using the Catmap package at R software [16] as previously described [15]. Briefly, OR were grouped by weighing individual OR by the inverse of their variance. Thus, the final effect OR and the 95% confidence interval (95%CI) were obtained by means of both random- and fixed-effects models. The fixed-effects model was used when the random-effects variance was less than or equal to zero and there was no heterogeneity, defined as *P* < 0.10 by the Cochran's (*Q*) test; otherwise, the random-effects model was chosen. Publication bias was evaluated by a sensitivity analysis.

### 2.3. Text Mining Approach

To find out the genetic similarity of the “HLA-A” and “Alzheimer's disease” concepts, we used the Anni software [17] because it uses the concept profile methodology that has proven to be effective in finding information in the form of associations in the biological domain [18], as previously described [19]. Briefly, we first mapped those concepts in the thesaurus of the software and built the concept profile for each one. These profiles corresponded to the weighted list made by all the genes mentioned in MedLine, so they were called genetic concept profiles (GCPs). To do this, we selected the 25.010 genes that belong to human beings from the thesaurus in Anni, and, then, we mined all the MedLine records that contained these genes in their text.

Next, we matched these two GCPs and analyzed the similarities between them. For this purpose, we obtained a cohesion score (CS) by using as an inclusive filter for matching the described 25.010 genes. To interpret the cohesion score we used a *P* value that gives the probability that the same CS or higher would be found in a random group of the same size. This *P* value was obtained by using the default parameter in Anni of 200 iterations. The contribution of each gene in the profile to the similarity between both GCPs was assessed in terms of percentage. The MedLine records that support a contribution higher than 0.1% to the similarity between GCPs were reviewed to verify that true genes, or the proteins they encode, were associated to the concepts “HLA-A” or “Alzheimer's disease.” Associations with ambiguous terms were eliminated.

### 2.4. Evaluation of Shared Genes by a Protein-Protein Interaction Network

To analyze which of the proteins encoded by the genes with the highest contribution to the similarity between GCPs truly interact, a network analysis was done with the genes that contributed at least 0.1% to the CS. For this purpose, the software, Genes2networks, was employed because it provides a reference network of experimentally known protein-protein interactions [20]. Then, in order to find tightly connected proteins, the settings that were used to build the network were (1) no filter for minimum number of references, (2) the maximum links per reference were four, (3) a maximum pathway length of two, and (4) a significant *z*-score of 2.5 of the intermediate nodes, which was calculated through a binomial proportions test, as previously described [19].

## 3. Results 

**Figure 1:**
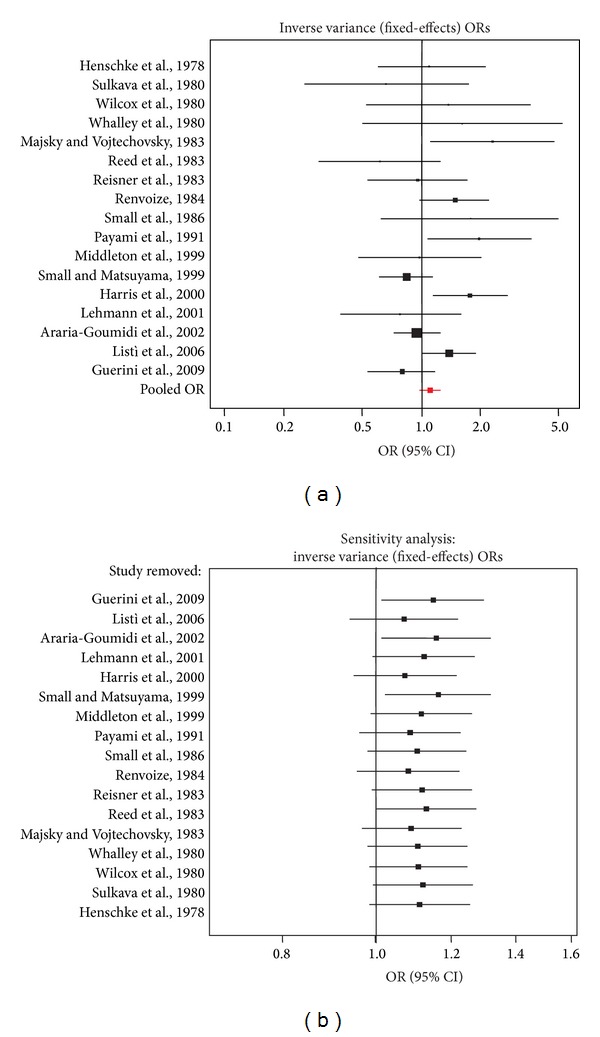
Forest plots of studies that relate HLA-A2 and AD. (a) Shows the effect summary OR (pooled OR) that takes into account all the studies. (b) Shows the pooled OR when each one of the studies was removed (sensitivity analysis).

From Europe, North-America and Asia, nineteen studies with data from case-control studies (2619 cases and 3878 controls) fitted the selection criteria; detailed information on the 185 articles that were excluded is given in the supplementary Table S1 in the Supplementary Material available online at http://dx.doi.org/10.1155/2014/791238. 17 of the included studies were from Caucasians [11, 12, 21–35] and 2 from Asians [13, 36]; see Table 1. Regarding the meta-analysis, the HLA-A2 did not have a specific behavior of risk or of protection with a pooled OR of 1.11, 95%CI: 0.98 to 1.24 (*QP* value 0.02). None of the articles included in the meta-analysis showed a significant OR lesser than one, but in contrast four studies showed a significant OR higher than one. In the same line, exclusion of one study by means of the sensitivity analysis led in some cases to significant risk behavior of the HLA-A2 but never to a significant protective behavior (Figure 1).

As it was recognized the cross-reactivity of the HLA-A2 antigen with HLA-A28, and that sera containing antibodies against A2 and A28 supertypic determinants are frequently found [37], we did a meta-analysis in Caucasians with the 8 studies that only used molecular techniques [11–13, 31–35]; see Table 1. The model showed similar unspecific results with a pooled OR of 1.03, 95%CI: 0.9 to 1.19 (*QP* value 0.03). With regard to the studies from Asia, the model also showed a very unspecific pooled OR than can be found between 0.84 and 2.16 (*QP* value 0.19).

Contrary to the nonconclusive results of the meta-analysis, the GCPs from both concepts AD and HLA-A were, not at random, genetically similar (CS *P* value 0.02). In addition to the* HLA-A*, 20 genes had a contribution higher than 0.1% to this similarity. As it was expected the weights of the genes involved in neuron remodeling and differentiation such as* APP* and* APLP2* were higher in the GCP of AD and the weights of the genes involved in immunity such as* HLA-DRB1* were higher in the GCP of HLA-A (Table 2).

**Figure 2:**
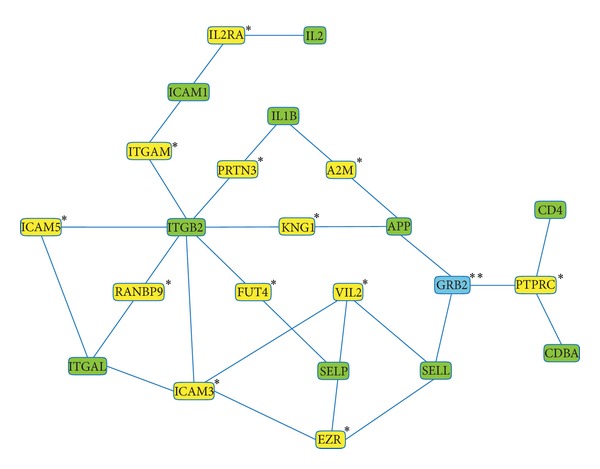
Interaction network of the proteins encoded by genes that contribute at least 0.1% to the cohesion score between HLA-A and AD. The nodes correspond to proteins encoded by the seed genes, to significant intermediary ones (indicated by one asterisk) and to a nonsignificant intermediary one (indicated by two asterisks).

Regarding the interaction analysis, proteins encoded from 10 of the 21 genes used as input were kept in the network, (Figure 2). Some genes shared by HLA-A and AD such as* APLP2*,* HLA-DRB1,* and* HFE* did not appear in the network despite their studied association with AD and/or HLA-A2 [8, 38, 39] and even the linkage disequilibrium with the HLA-A gene [40]. This could have been because of the strict threshold, a maximum pathway length of two, established to avoid weak interactions. Furthermore, the network had 13 intermediary nodes, 12 significant with a *z*-score above the cutoff of 2.5 (Table 3), thus indicating that the seed genes encode proteins that had strong and specific interactions.

In the graph, we found out two subnetworks (Figure 2): The first was made up of APP (involved in remodeling, differentiation, and apoptosis of neurons), ICAM1, ITGB2, ITGAL, SELP and SELL (involved in leukocyte adhesion, rolling over vascular surfaces, and transendotelial migration), and IL2 and IL1B (involved in T-cell proliferation, activation, and inflammation) and the second subnetwork was made up of CD4 and CD8A (involved in HLA classes I and II antigen presentation). These two subnetworks were connected by the growth factor receptor-bound protein (GRB2), the unique intermediate node that had a nonsignificant *z*-score due to low specificity because of its many links (Table 3), in other words, because it is a molecule involved in many cellular processes [41].

## 4. Discussion

Despite having new studies with big samples and homogeneous criteria for inclusion of AD patients (i.e. NINCDS-ADRDA) compared to the meta-analysis that associated HLA-A2 and AD more than ten years ago [12], we did not find conclusive results by this classical approach. The HLA-A2 showed to be a mild risk factor of AD with significant results only in some populations, thus suggesting that there are processes that influence this relationship. In contrast, the in silico approach retrieved nonrandomly shared genes by the concepts of HLA-A and AD (*P* = 0.02), that additionally encode truly interacting proteins. Proteins encoded by* APP*,* ICAM-1*,* ITGB2*,* ITGAL*,* SELP*,* SELL*,* IL2*,* IL1B*,* CD4,* and* CD8A* interact and were statistically and experimentally related to both concepts: HLA-A and AD. The network of interacting proteins highlighted specific processes, thus assisting to point out relevant pathogenic pathways that linked immunity to AD. Immune processes such as leukocyte adhesion and transendothelial migration, peptide presentation, and T-cell activation and proliferation were linked to processes traditionally involved in the pathogenesis of AD such as remodeling and apoptosis of neurons.

The results of our meta-analysis point out the importance of finding out relevant gene networks than can influence the relationship between HLA-A and AD and are in the same line with a previous analysis of the HLA-B, another gene of the same complex genomic region. In Oxford, researchers from UK confirmed in 2006 the association between HLA-B7 and AD, which was previously found in other people from the same city in 2001. However, this association was not found in populations from Cambridge, UK, and Montreal, Canada in spite the fact that all were of Caucasian origin. That is why, it was suggested a geographical specificity that could be due to different interactions with other processes of environmental, genetic or epigenetic origin [42].

Regarding the highlighted processes by our analysis of gene networks, it has been observed alterations of endothelial regulation in AD. IL1A, IL1B, IL2, IL8, IFN*γ*, and TNF*α* have been found to be associated with senile plaques. Some of them, IL8 and IFN*γ* were also significantly increased in plasma. Abnormal secretion of cytokines due to immune activation may impair the regulation of endothelial cells and induce altered pathways of adhesion molecules. There have been observed lower levels of P-selectin and L-selectin in AD and lowest in patients with the highest cognitive decline, thus leading to impaired regulation of both endothelial function and leukocyte migration [43]. With this landscape, infections at the level of the vasculature may be a key initiating factor in the pathogenesis of neurodegenerative diseases such as sporadic AD. Some observations have shown that* C. pneumoniae *infection stimulates transendothelial entry of monocytes through human brain endothelial cells (HBMECs). This entry is facilitated by the upregulation of VCAM-1 and ICAM-1 on HBMECs and a corresponding increase of ITGB2-ITGAL (LFA-1), VLA-4, and ITGB2-ITGAM (MAC-1) on monocytes [44].

Another important process is the antigen presentation as it has been demonstrated the highly immunogenic properties of one specific HLA class II allele in a model of AD. It was observed that Abeta was effectively cleared from the brain parenchyma and brain microglial activation was reduced in long-term therapeutic immunization of an AD mouse model bearing the DRB1∗1501 allele [45]. Regarding to HLA-A2, it is a HLA class I protein that not only plays roles in the initiation of antigen receptor signaling, but is also expressed in neurons throughout the CNS. Neuronal HLA class I is upregulated after exposure to cytokines and functions as a mediator of synaptic plasticity during development of the visual system. Additional studies suggest that HLA class I may regulate the ability of neurons to maintain synapses. HLA class I mediated signaling has been studied, and it was observed that specifically HLA-A2 is substrate for the Alzheimer's disease-associated presenilin-1/gamma-secretase [46].

All in all, there is important evidence of the association between the described processes. A Genome Wide Association study found a significant single nucleotide polymorphism associated with AD within* GAB2*, which encodes “GRB2-associated binding protein 2” (Gab2). Gab2 binds GRB2 that also binds tau, APP, presenilin-1, and presenilin-2. Consequently, Gab2 could conceivably modulate APP processing and/or tau phosphorylation via its interaction with GRB2 [47]. Additionally, GRB2 is a known adapter with a recently described role in antigen receptor signaling as well as lymphocyte development [41].

## 5. Conclusion

Our review gives support to the immune involvement in AD. However, we not only find out a network of interacting proteins that links neurodegenerative to immune processes but that also gives hints for further research such as infectious diseases that alter the endothelial regulation as possible starting factor in AD, the role of GRB2 as a molecule that links antigen presentation with neuronal processes or the HLA-A2 role in the typical synaptic loss of AD. Thus, taking into account the described findings and the current overwhelming amount of data it seems highly advisable to combine in silico techniques with classical approaches such as systematic reviews or meta-analyses to find useful information.

## Supplementary Material

Description of the causes of exclusion for each one of the studies not included in the meta-analysis.

## Figures and Tables

**Table 1:** Description of the included articles.

Study	Country/population	Cases/controls	Typing/technique	Diagnostic criteria	Reference
Henschke et al., 1978	Canada	34/239	Lymphotoxicity	Exclusion of other causes of dementia	[21]
Sulkava et al., 1980	Finland	32/35	Lymphotoxicity	Exclusion of other causes of dementia	[22]
Wilcox et al., 1980	United Kingdom	18/342	Lymphotoxicity	Exclusion of other causes of dementia	[23]
Whalley et al., 1980	United Kingdom	14/64	Lymphotoxicity	Exclusion of other causes of dementia	[24]
Majsky and Vojtechovsky, 1983	Czechoslovakia	38/301	Lymphotoxicity	Exclusion of other causes of dementia-Haschinsky	[25]
Reed et al., 1983	United States	44/100	Lymphotoxicity	Exclusion of other causes of dementia	[26]
Reisner et al., 1983	United States	52/305	Amos modified method	Histopathological confirmation	[27]
Renvoize, 1984	United Kingdom	124/458	Lympho-toxicity	Exclusion of other causes of dementia	[28]
Endo et al., 1986	Japan	122/66	Lympho-toxicity	DSM III	[36]
Small and Matsuyama, 1986	United States	36/25	Lympho-toxicity	DSM III	[29]
Payami et al., 1991	United States	54/263	Lymphotoxicity	NINCDS-ADRDA	[30]
Middleton et al., 1999	United Kingdom	95/45	PCR SSOP	Histopathological confirmation	[31]
Small et al., 1999	United States	479/233	PCR SSP	NINCDS-ADRDA	[32]
Harris et al., 2000	United Kingdom	178/161	PCR SSP	NINCDS-ADRDA	[12]
Lehmann et al., 2001	United Kingdom	55/73	PCR SSP	Histopathological confirmation CERAD	[11]
Araria-Goumidi et al., 2002	France	451/477	Duplex-PCR	DSM III and NINCDS-ADRDA	[33]
Listì et al., 2006	Italy	460/266	PCR SSP	NINCDS-ADRDA	[34]
Ma et al., 2008	China	160/167	SBT	NINCDS-ADRDA	[13]
Guerini et al., 2009	Italy	173/258	PCR SSP	NINCDS-ADRDA	[35]

AD: Alzheimer's disease, PCR SSOP: Polymerase chain reaction and sequence specific oligonucleotide probe, PCR SSP: Polymerase chain reaction and sequence specific primers, SBT: Sequence based typing, DSM: Diagnostic and Statistical Manual, NINCDS-ADRDA: National Institute of Neurological and Communicative Disorders and Stroke and the Alzheimer's Disease and Related Disorders Association, CERAD: Consortium to Establish a Registry for Alzheimer's Disease.

**Table 2:** Genes with a contribution higher than 0.1% to the similarity between AD and HLA-A.

Gene	Percentage	Weight in AD	Weight in HLA-A
*HLA-A *	38.870	1.257*E* − 6	3.990*E* − 2
*APLP2 *	21.170	4.900*E* − 3	5.546*E* − 6
*APP *	17.115	7.000*E* − 3	3.176*E* − 6
*HLA-DRB1 *	5.576	3.333*E* − 6	2.200*E* − 3
*CD8A *	3.116	1.434*E* − 5	3.000*E* − 4
*ICAM1 *	2.115	5.694*E* − 6	5.000*E* − 4
*HLA-B *	1.904	1.067*E* − 6	2.300*E* − 3
*HLA-C *	1.721	1.472*E* − 6	1.500*E* − 3
*CD4 *	0.611	2.812*E* − 5	2.807*E* − 5
*IFNG *	0.391	6.326*E* − 6	7.995*E* − 5
*HFE *	0.223	2.891*E* − 6	9.967*E* − 5
*IL1B *	0.176	2.309*E* − 5	9.881*E* − 6
*IL2 *	0.170	8.815*E* − 6	2.494*E* − 5
*TYR *	0.165	2.515*E* − 6	8.474*E* − 5
*WT1 *	0.147	1.599*E* − 6	1.000*E* − 4
*SELL *	0.138	1.748*E* − 6	1.000*E* − 4
*ITGB2 *	0.135	4.105*E* − 6	4.252*E* − 5
*ITGAL *	0.128	2.750*E* − 6	6.013*E* − 5
*CD80 *	0.124	4.628*E* − 6	3.470*E* − 5
*CD86 *	0.123	3.332*E* − 6	4.798*E* − 5
*SELP *	0.113	2.683*E* − 6	5.478*E* − 5

Cohesion score *P* value 0.02.

**Table 3:** Significance of intermediates sorted by *z*-score.

Node name	Links	Links in background	Links to seed	Links in subnetwork	*z*-score
FUT4	3	11429	2	29	22.86
ICAM5	4	11429	2	29	19.77
PRTN3	9	11429	2	29	13.10
IL2RA	22	11429	3	29	12.47
ICAM3	10	11429	2	29	12.41
VIL2	32	11429	3	29	10.25
ITGAM	15	11429	2	29	10.06
EZR	34	11429	3	29	9.93
KNG1	22	11429	2	29	8.23
RANBP9	22	11429	2	29	8.23
A2M	24	11429	2	29	7.86
PTPRC	35	11429	2	29	6.42
GRB2	196	11429	2	29	2.13

## Abbreviations

95%CI: 95% confidence interval
Abeta: Beta-amyloid
AD: Alzheimer disease
APP: Amyloid precursor protein
CERAD: The Consortium to Establish a Registry for Alzheimer's Disease
CNS: Central nervous system
CS: Cohesion score
DSM: Diagnostic and Statistical Manual
GCPs: Genetic concept profiles
HBMECs: Human brain endothelial cells
HLA: Human leukocyte antigen
NINCDS-ADRDA: National Institute of Neurological and Communicative Disorders and Stroke and the Alzheimer's Disease and Related Disorders Association
OR: Odds ratio.
