# Supplementary material for: Alzheimer's Disease and HLA-A2: Linking Neurodegenerative to Immune Processes through an In Silico Approach
Source: Biomed Res Int. 2014 Aug 17;2014:791238. doi: 10.1155/2014/791238 (PMC4150521; doi:10.1155/2014/791238)
Supplement: Supplementary file 1 — Description of the causes of exclusion for each one of the studies not included in the meta-analysis. [file 791238.f1.pdf]

Supplemental materials

Supplementary Table 1. Excluded articles in the manuscript HLA-A2 and Alzheimer´s Disease; linking Immune to Neurodegenerative processes.

Cause of Exclusion	Supplementary Reference
No association between HLA and AD in title/abstract	(1)
No association between HLA and AD in title/abstract.	(2)
No association between HLA and AD in title/abstract	(3)
Not a case-control study	(4)
No association between HLA and AD in title/abstract	(5)
Not a case-control study	(6)
No association between HLA and AD in title/abstract	(7)
No association between HLA and AD in title/abstract	(8)
No association between HLA and AD in title/abstract	(9)
No association between HLA and AD in title/abstract	(10)
No association between HLA and AD in title/abstract	(11)
No association between HLA and AD in title/abstract	(12)
No association between HLA and AD in title/abstract	(13)
No association between HLA and AD in title/abstract	(14)
No association between HLA and AD in title/abstract	(15)
No enough HLA-A2 data to calculate reliable OR	(16)
No association between HLA and AD in title/abstract	(17)
No association between HLA and AD in title/abstract	(18)
No association between HLA and AD in title/abstract	(19)
No association between HLA and AD in title/abstract	(20)
No association between HLA and AD in title/abstract	(21)
No association between HLA and AD in title/abstract	(22)
No association between HLA and AD in title/abstract	(23)
No association between HLA and AD in title/abstract	(24)
No association between HLA and AD in title/abstract	(25)
No association between HLA and AD in title/abstract	(26)
No association between HLA and AD in title/abstract	(27)
No association between HLA and AD in title/abstract	(28)

No association between HLA and AD in title/abstract	(29)
No association between HLA and AD in title/abstract	(30)
No association between HLA and AD in title/abstract	(31)
No association between HLA and AD in title/abstract	(32)
No association between HLA and AD in title/abstract	(33)
No association between HLA and AD in title/abstract	(34)
No association between HLA and AD in title/abstract	(35)
No association between HLA and AD in title/abstract	(36)
Not a case-control study	(37)
No association between HLA and AD in title/abstract	(38)
No association between HLA and AD in title/abstract	(39)
No association between HLA and AD in title/abstract	(40)
No association between HLA and AD in title/abstract	(41)
No association between HLA and AD in title/abstract	(42)
No association between HLA and AD in title/abstract	(43)
No association between HLA and AD in title/abstract	(44)
No association between HLA and AD in title/abstract	(45)
No association between HLA and AD in title/abstract	(46)
No enough HLA-A2 data to calculate reliable OR	(47)
No association between HLA and AD in title/abstract	(48)
No association between HLA and AD in title/abstract	(49)
No association between HLA and AD in title/abstract	(50)
No enough HLA-A2 data to calculate reliable OR	(51)
No association between HLA and AD in title/abstract	(52)
No association between HLA and AD in title/abstract	(53)
No association between HLA and AD in title/abstract	(54)
No association between HLA and AD in title/abstract	(55)
No association between HLA and AD in title/abstract	(56)
Not a case-control study	(57)
No association between HLA and AD in title/abstract	(58)
No association between HLA and AD in title/abstract	(59)
No association between HLA and AD in title/abstract	(60)
No association between HLA and AD in title/abstract	(61)
No association between HLA and AD in title/abstract	(62)
No association between HLA and AD in title/abstract	(63)
Not a case-control study	(64)

No association between HLA and AD in title/abstract	(65)
No association between HLA and AD in title/abstract	(66)
No association between HLA and AD in title/abstract	(67)
No association between HLA and AD in title/abstract	(68)
No association between HLA and AD in title/abstract	(69)
No association between HLA and AD in title/abstract	(70)
Not a case-control study	(71)
No association between HLA and AD in title/abstract	(72)
No association between HLA and AD in title/abstract	(73)
No association between HLA and AD in title/abstract	(74)
No association between HLA and AD in title/abstract	(75)
No association between HLA and AD in title/abstract	(76)
No association between HLA and AD in title/abstract	(77)
No association between HLA and AD in title/abstract	(78)
No association between HLA and AD in title/abstract	(79)
No association between HLA and AD in title/abstract	(80)
No association between HLA and AD in title/abstract	(81)
No association between HLA and AD in title/abstract	(82)
No association between HLA and AD in title/abstract	(83)
No association between HLA and AD in title/abstract	(84)
No association between HLA and AD in title/abstract	(85)
No enough HLA-A2 data to calculate reliable OR	(86)
No enough HLA-A2 data to calculate reliable OR	(87)
No association between HLA and AD in title/abstract	(88)
No association between HLA and AD in title/abstract	(89)
No association between HLA and AD in title/abstract	(90)
No association between HLA and AD in title/abstract	(91)
No association between HLA and AD in title/abstract	(92)
Etiology of cases related to *APP*, *PS1*, or *PS2* mutations	(93)
No association between HLA and AD in title/abstract	(94)
No enough HLA-A2 data to calculate reliable OR	(95)
No association between HLA and AD in title/abstract	(96)
No enough HLA-A2 data to calculate reliable OR	(97)
No association between HLA and AD in title/abstract	(98)
No enough HLA-A2 data to calculate reliable OR	(99)
No association between HLA and AD in title/abstract	(100)

No association between HLA and AD in title/abstract	(101)
No association between HLA and AD in title/abstract	(102)
No association between HLA and AD in title/abstract	(103)
No association between HLA and AD in title/abstract	(104)
Not a case-control study	(105)
No enough HLA-A2 data to calculate reliable OR	(106)
No association between HLA and AD in title/abstract	(107)
No association between HLA and AD in title/abstract	(108)
No association between HLA and AD in title/abstract	(109)
No association between HLA and AD in title/abstract	(110)
No association between HLA and AD in title/abstract	(111)
Not a case-control study	(112)
No association between HLA and AD in title/abstract	(113)
No association between HLA and AD in title/abstract	(114)
No association between HLA and AD in title/abstract	(115)
No association between HLA and AD in title/abstract	(116)
No association between HLA and AD in title/abstract	(117)
No association between HLA and AD in title/abstract	(118)
No association between HLA and AD in title/abstract	(119)
No enough HLA-A2 data to calculate reliable OR	(120)
No association between HLA and AD in title/abstract	(121)
No association between HLA and AD in title/abstract	(122)
No enough HLA-A2 data to calculate reliable OR	(123)
No association between HLA and AD in title/abstract	(124)
No association between HLA and AD in title/abstract	(125)
No association between HLA and AD in title/abstract	(126)
Not a case-control study	(127)
No association between HLA and AD in title/abstract	(128)
No association between HLA and AD in title/abstract	(129)
No association between HLA and AD in title/abstract	(130)
No association between HLA and AD in title/abstract	(131)
No association between HLA and AD in title/abstract	(132)
Not a case-control study	(133)
No association between HLA and AD in title/abstract	(134)
No association between HLA and AD in title/abstract	(135)
No association between HLA and AD in title/abstract	(136)

No enough HLA-A2 data to calculate reliable OR	(137)
No enough HLA-A2 data to calculate reliable OR	(138)
No association between HLA and AD in title/abstract	(139)
No association between HLA and AD in title/abstract	(140)
No association between HLA and AD in title/abstract	(141)
Same subjects as in reference (142)	(143)
Not a case-control study	(144)
No enough HLA-A2 data to calculate reliable OR	(145)
No association between HLA and AD in title/abstract	(146)
Not a case-control study	(147)
No association between HLA and AD in title/abstract	(148)
Not a case-control study	(149)
No data to calculate the OR of HLA-A2 between cases and controls	(150)
No enough HLA-A2 data to calculate reliable OR	(151)
No association between HLA and AD in title/abstract	(152)
Not a case-control study	(153)
Same subjects as in reference (142)	(154)
No association between HLA and AD in title/abstract	(155)
Not a case-control study	(156)
No association between HLA and AD in title/abstract	(157)
Not a case-control study	(158)
No association between HLA and AD in title/abstract	(159)
No association between HLA and AD in title/abstract	(160)
Not a case-control study	(161)
No association between HLA and AD in title/abstract	(162)
Not a case-control study	(163)
No association between HLA and AD in title/abstract	(164)
No association between HLA and AD in title/abstract	(165)
No association between HLA and AD in title/abstract	(166)
No association between HLA and AD in title/abstract	(167)
No enough HLA-A2 data to calculate reliable OR	(168)
Not a case-control study	(169)
No association between HLA and AD in title/abstract	(170)
No association between HLA and AD in title/abstract	(171)
No association between HLA and AD in title/abstract	(172)

No association between HLA and AD in title/abstract	(173)
Not a case-control study	(174)
No association between HLA and AD in title/abstract	(175)
Not a case-control study	(176)
Not a case-control study	(177)
No association between HLA and AD in title/abstract	(178)
No association between HLA and AD in title/abstract	(179)
No association between HLA and AD in title/abstract	(180)
Not a case-control study	(181)
Not a case-control study	(182)
No association between HLA and AD in title/abstract	(183)
Same subjects as in reference 3	(184)
No association between HLA and AD in title/abstract	(185)

OR: Odds ratio, HLA: Human leukocyte antigen, AD: Alzheimer´s disease, *APP:* amyloid precursor protein, *PS1:* presenilin-1, *PS2:* presenilin-2

1. Schrier RD, Gupta S, Riggs P, Cysique LA, Letendre S, Jin H, et al. The influence of HLA on HIV-associated neurocognitive impairment in Anhui, China. PLoS One. 2012;7(5):e32303.
2. Gulyas B, Pavlova E, Kasa P, Gulya K, Bakota L, Varszegi S, et al. Activated MAO-B in the brain of Alzheimer patients, demonstrated by [11C]-L-deprenyl using whole hemisphere autoradiography. Neurochem Int. 2011 Jan;58(1):60-8.
3. Hoozemans JJ, Rozemuller AJ, van Haastert ES, Eikelenboom P, van Gool WA. Neuroinflammation in Alzheimer's disease wanes with age. J Neuroinflammation. 2011;8:171.
4. Nemirovsky A, Fisher Y, Baron R, Cohen IR, Monsonego A. Amyloid beta-HSP60 peptide conjugate vaccine treats a mouse model of Alzheimer's disease. Vaccine. 2011 May 23;29(23):4043-50.
5. Zotova E, Holmes C, Johnston D, Neal JW, Nicoll JA, Boche D. Microglial alterations in human Alzheimer's disease following Abeta42 immunization. Neuropathol Appl Neurobiol. 2011 Aug;37(5):513-24.
6. Boiocchi C, Maggioli E, Ricevuti G, Gagliardi S, Cereda C, Cuccia M. An haplotypic study of HLA class III gene polymorphisms in Alzheimer disease. Tissue Antigens. 2011;77(5):511.
7. Cardoso CC, Pereira AC, De Sales Marques C, Moraes MO. Leprosy susceptibility: Genetic variations regulate innate and adaptive immunity, and disease outcome. Future Microbiology. 2011;6(5):533-49.
8. Marin O, Gleeson JG. Function follows form: Understanding brain function from a genetic perspective. Current Opinion in Genetics and Development. 2011;21(3):237-9.
9. Liu X, Cheng R, Verbitsky M, Kisselev S, Browne A, Mejia-Sanatana H, et al. Genome-wide association study identifies candidate genes for Parkinson's disease in an Ashkenazi Jewish population. BMC Med Genet. 2011;12:104.
10. Leung E, Guo L, Bu J, Maloof M, Khoury JE, Geula C. Microglia activation mediates fibrillar amyloid-β toxicity in the aged primate cortex. Neurobiology of Aging. 2011;32(3):387-97.
11. Huang X, Wang J, Cui L, Zou X, Zhang Y. Recombinant GST-I-A beta 28-induced efficient serum antibody against A beta 42. J Neurosci Methods. 2010 Jan 30;186(1):52-9.
12. Xia Z, Chibnik LB, Glanz BI, Liguori M, Shulman JM, Tran D, et al. A putative Alzheimer's disease risk allele in PCK1 influences brain atrophy in multiple sclerosis. PLoS One. 2010;5(11):e14169.
13. Grunhage F, Nattermann J. Viral hepatitis: Human genes that limit infection. Best Practice and Research: Clinical Gastroenterology. 2010;24(5):709-23.
14. An L, Sato H, Konishi Y, Walker DG, Beach TG, Rogers J, et al. Expression and localization of lactotransferrin messenger RNA in the cortex of Alzheimer's disease. Neurosci Lett. 2009 Mar 20;452(3):277-80.
15. Correia AP, Pinto JP, Dias V, Mascarenhas C, Almeida S, Porto G. CAT53 and HFE alleles in Alzheimer's disease: a putative protective role of the C282Y HFE mutation. Neurosci Lett. 2009 Jul 3;457(3):129-32.
16. Guerini FR, Tinelli C, Calabrese E, Agliardi C, Zanzottera M, De Silvestri A, et al. HLA-A*01 is associated with late onset of Alzheimer's disease in Italian patients. Int J Immunopathol Pharmacol. 2009 Oct-Dec;22(4):991-9.
17. Kellner A, Matschke J, Bernreuther C, Moch H, Ferrer I, Glatzel M. Autoantibodies against beta-amyloid are common in Alzheimer's disease and help control plaque burden. Ann Neurol. 2009 Jan;65(1):24-31.
18. McIntyre JA, Wagenknecht DR, Ramsey CJ. Redox-reactive antiphospholipid antibody differences between serum from Alzheimer's patients and age-matched controls. Autoimmunity. 2009;42(8):646-52.

19. Quiroga I, Lehmann DJ, Barnardo MC, Fuggle S, Cortina-Borja M, Warden DR, et al. Association study of MICA and MICB in Alzheimer's disease. Tissue Antigens. 2009 Sep;74(3):241-3.
20. Roses AD. The medical and economic roles of pipeline pharmacogenetics: Alzheimer's disease as a model of efficacy and HLA-B(*)5701 as a model of safety. Neuropsychopharmacology. 2009 Jan;34(1):6-17.
21. Ryu JK, Cho T, Choi HB, Wang YT, McLarnon JG. Microglial VEGF receptor response is an integral chemotactic component in Alzheimer's disease pathology. J Neurosci. 2009 Jan 7;29(1):3-13.
22. Shimizu H, Ghazizadeh M, Sato S, Oguro T, Kawanami O. Interaction between beta-amyloid protein and heparan sulfate proteoglycans from the cerebral capillary basement membrane in Alzheimer's disease. J Clin Neurosci. 2009 Feb;16(2):277-82.
23. Walker DG, Dalsing-Hernandez JE, Campbell NA, Lue LF. Decreased expression of CD200 and CD200 receptor in Alzheimer's disease: a potential mechanism leading to chronic inflammation. Exp Neurol. 2009 Jan;215(1):5-19.
24. Zhang R, Miller RG, Gascon R, Champion S, Katz J, Lancero M, et al. Circulating endotoxin and systemic immune activation in sporadic amyotrophic lateral sclerosis (sALS). J Neuroimmunol. 2009 Jan 3;206(1-2):121-4.
25. Zota V, Nemirovsky A, Baron R, Fisher Y, Selkoe DJ, Altmann DM, et al. HLA-DR alleles in amyloid beta-peptide autoimmunity: a highly immunogenic role for the DRB1*1501 allele. J Immunol. 2009 Sep 1;183(5):3522-30.
26. Ciaramella A, Sanarico N, Bizzoni F, Moro ML, Salani F, Scapigliati G, et al. Amyloid (beta) peptide promotes differentiation of pro-inflammatory human myeloid dendritic cells. Neurobiology of Aging. 2009;30(2):210-21.
27. Kalman B, Vitale E. Structural chromosomal variations in neurological diseases. Neurologist. 2009;15(5):245-53.
28. Shepherd CE, Gregory G, Warden L, Halliday G. Oligomeric A(beta)40 species accumulate in the toxic plaques in Alzheimer's disease. Alzheimer's and Dementia. 2009;5(4):107.
29. Xue SR, Xu DH, Yang XX, Dong WL. Alterations in lymphocyte subset patterns and co-stimulatory molecules in patients with Alzheimer disease. Chin Med J (Engl). 2009 Jun 20;122(12):1469-72.
30. Gnjec A, D'Costa KJ, Laws SM, Hedley R, Balakrishnan K, Taddei K, et al. Association of alleles carried at TNFA -850 and BAT1 -22 with Alzheimer's disease. J Neuroinflammation. 2008;5:36.
31. Gouw AA, Seewann A, Vrenken H, van der Flier WM, Rozemuller JM, Barkhof F, et al. Heterogeneity of white matter hyperintensities in Alzheimer's disease: post-mortem quantitative MRI and neuropathology. Brain. 2008 Dec;131(Pt 12):3286-98.
32. Lopes KO, Sparks DL, Streit WJ. Microglial dystrophy in the aged and Alzheimer's disease brain is associated with ferritin immunoreactivity. Glia. 2008 Aug 1;56(10):1048-60.
33. Maekawa M, Watanabe Y. Epigenetics: relations to disease and laboratory findings. Curr Med Chem. 2007;14(25):2642-53.
34. McIntyre JA, Chapman J, Shavit E, Hamilton RL, Dekosky ST. Redox-reactive autoantibodies in Alzheimer's patients' cerebrospinal fluids: preliminary studies. Autoimmunity. 2007 Jul;40(5):390-6.
35. McIntyre JA, Hamilton RL, DeKosky ST. Redox-reactive autoantibodies in cerebrospinal fluids. Ann N Y Acad Sci. 2007 Aug;1109:296-302.
36. Verwer RWH, Sluiter AA, Balesar RA, Baayen JC, Noske DP, Dirven CMF, et al. Mature astrocytes in the adult human neocortex express the early neuronal marker doublecortin. Brain. 2007;130(12):3321-35.
37. Alves C, Veiga S, Souza T, Toralles MB, Da Silva-Bacellar AL. The role of the human histocompatibility antigens in the pathogenesis of neurological disorders. Revista de Neurologia. 2007;44(5):298-302.

38. Amador-Ortiz C, Ahmed Z, Zehr C, Dickson DW. Hippocampal sclerosis dementia differs from hippocampal sclerosis in frontal lobe degeneration. Acta Neuropathol. 2007 Mar;113(3):245-52.
39. Carey BW, Kim DY, Kovacs DM. Presenilin/gamma-secretase and alpha-secretase-like peptidases cleave human MHC Class I proteins. Biochem J. 2007 Jan 1;401(1):121-7.
40. Chiappelli M, Tumini E, Porcellini E, Licastro F. Impaired regulation of immune responses in cognitive decline and Alzheimer's disease: lessons from genetic association studies. Expert Rev Neurother. 2006 Sep;6(9):1327-36.
41. McLarnon JG, Ryu JK, Walker DG, Choi HB. Upregulated expression of purinergic P2X(7) receptor in Alzheimer disease and amyloid-beta peptide-treated microglia and in peptide-injected rat hippocampus. J Neuropathol Exp Neurol. 2006 Nov;65(11):1090-7.
42. O'Brien TA, Tiedemann K, Vowels MR. No longer a biological waste product: umbilical cord blood. Med J Aust. 2006 Apr 17;184(8):407-10.
43. Wurtman RJ. Narcolepsy and the hypocretins. Metabolism. 2006 Oct;55(10 Suppl 2):S36-9.
44. Xiang Z, Haroutunian V, Ho L, Purohit D, Pasinetti GM. Microglia activation in the brain as inflammatory biomarker of Alzheimer's disease neuropathology and clinical dementia. Dis Markers. 2006;22(1-2):95-102.
45. Youdim MB. The path from anti Parkinson drug selegiline and rasagiline to multifunctional neuroprotective anti Alzheimer drugs ladostigil and m30. Curr Alzheimer Res. 2006 Dec;3(5):541-50.
46. Payton A, van den Boogerd E, Davidson Y, Gibbons L, Ollier W, Rabbitt P, et al. Influence and interactions of cathepsin D, HLA-DRB1 and APOE on cognitive abilities in an older non-demented population. Genes Brain Behav. 2006;5 Suppl 1:23-31.
47. Lehmann DJ, Barnardo MC, Fuggle S, Quiroga I, Sutherland A, Warden DR, et al. Replication of the association of HLA-B7 with Alzheimer's disease: a role for homozygosity? J Neuroinflammation. 2006;3:33.
48. Kutzler MA, Cao C, Bai Y, Dong H, Choe PY, Saulino V, et al. Mapping of immune responses following wild-type and mutant ABeta42 plasmid or peptide vaccination in different mouse haplotypes and HLA Class II transgenic mice. Vaccine. 2006 May 22;24(21):4630-9.
49. Agadjanyan MG, Ghochikyan A, Petrushina I, Vasilevko V, Movsesyan N, Mkrtichyan M, et al. Prototype Alzheimer's disease vaccine using the immunodominant B cell epitope from beta-amyloid and promiscuous T cell epitope pan HLA DR-binding peptide. J Immunol. 2005 Feb 1;174(3):1580-6.
50. D'Andrea MR. Evidence that immunoglobulin-positive neurons in Alzheimer's disease are dying via the classical antibody-dependent complement pathway. Am J Alzheimers Dis Other Demen. 2005 May-Jun;20(3):144-50.
51. Fiala M, Lin J, Ringman J, Kermani-Arab V, Tsao G, Patel A, et al. Ineffective phagocytosis of amyloid-beta by macrophages of Alzheimer's disease patients. J Alzheimers Dis. 2005 Jun;7(3):221-32; discussion 55-62.
52. Raha-Chowdhury R, Andrews SR, Gruen JR. CAT 53: a protein phosphatase 1 nuclear targeting subunit encoded in the MHC Class I region strongly expressed in regions of the brain involved in memory, learning, and Alzheimer's disease. Brain Res Mol Brain Res. 2005 Jul 29;138(1):70-83.
53. Wiendl H, Feger U, Mittelbronn M, Jack C, Schreiner B, Stadelmann C, et al. Expression of the immune-tolerogenic major histocompatibility molecule HLA-G in multiple sclerosis: implications for CNS immunity. Brain. 2005 Nov;128(Pt 11):2689-704.
54. Youdim MBH, Fridkin M, Zheng H. Bifunctional drug derivatives of MAO-B inhibitor rasagiline and iron chelator VK-28 as a more effective approach to treatment of brain ageing and ageing neurodegenerative diseases. Mechanisms of Ageing and Development. 2005;126(2):317-26.
55. Kopp N. A biological definition of the human person. Ethique et Sante. 2005;2(4):207-14.

56. Lao JI, Montoriol C, Morer I, Beyer K. Genetic contribution to aging: deleterious and helpful genes define life expectancy. Ann N Y Acad Sci. 2005 Dec;1057:50-63.
57. Hosford DA, Lai EH, Riley JH, Xu CF, Danoff TM, Roses AD. Pharmacogenetics to predict drug-related adverse events. Toxicol Pathol. 2004 Mar-Apr;32 Suppl 1:9-12.
58. Lombardi VR, Fernandez-Novoa L, Etcheverria I, Seoane S, Cacabelos R. Association between APOE epsilon4 allele and increased expression of CD95 on T cells from patients with Alzheimer's disease. Methods Find Exp Clin Pharmacol. 2004 Sep;26(7):523-9.
59. Roberts ES, Masliah E, Fox HS. CD163 identifies a unique population of ramified microglia in HIV encephalitis (HIVE). J Neuropathol Exp Neurol. 2004 Dec;63(12):1255-64.
60. Simmons DL, Botting RM, Hla T. Cyclooxygenase isozymes: the biology of prostaglandin synthesis and inhibition. Pharmacol Rev. 2004 Sep;56(3):387-437.
61. Shepherd CE, Piguet O, Broe GA, Creasey H, Waite LM, Brooks WS, et al. Histocompatibility antigens, aspirin use and cognitive performance in non-demented elderly subjects. J Neuroimmunol. 2004 Mar;148(1-2):178-82.
62. Frey J. [Pheromones: an underestimated communication signal in humans]. Ann Biol Clin (Paris). 2003 May-Jun;61(3):275-8.
63. Lampe JB, Gossrau G, Kempe A, Fussel M, Schwurack K, Schroder R, et al. Analysis of HLA class I and II alleles in sporadic inclusion-body myositis. Journal of Neurology. 2003;250(11):1313-7.
64. Nourhashemi F, Gillette-Guyonnet S, Fort M, Andrieu S, Abbal M, Albarede JL, et al. [Association between the A2 allele of the HLA system and age at onset of Alzheimer's disease]. Presse Med. 2003 Feb 1;32(4):158-61.
65. Eymard B. [Polymyositis, dermatomyositis and inclusion body myositis, nosological aspects]. Presse Med. 2003 Oct 25;32(35):1656-67.
66. Middleton PG, Norden J, Cullup H, Cavet J, Jackson GH, Taylor PR, et al. Oestrogen receptor alpha gene polymorphism associates with occurrence of graft-versus-host disease and reduced survival in HLA-matched sib-allo BMT. Bone Marrow Transplant. 2003 Jul;32(1):41-7.
67. Schmidt S, Marrosu GM, Kolsch H, Haase CG, Ferenczik S, Sokolowski P, et al. Genetic variations and humoral immune responses to myelin oligodendroglia glycoprotein in adult phenotypes of X-linked adrenoleukodystrophy. J Neuroimmunol. 2003 Feb;135(1-2):148-53.
68. Lee YB, Nagai A, Kim SU. Cytokines, chemokines, and cytokine receptors in human microglia. J Neurosci Res. 2002 Jul 1;69(1):94-103.
69. Wierzba-Bobrowicz T, Gwiazda E, Kosno-Kruszewska E, Lewandowska E, Lechowicz W, Bertrand E, et al. Morphological analysis of active microglia--rod and ramified microglia in human brains affected by some neurological diseases (SSPE, Alzheimer's disease and Wilson's disease). Folia Neuropathol. 2002;40(3):125-31.
70. Consolandi C, Bordoni R, Castiglioni B, Frosini A, Mezzelani A, Rizzi E, et al. Investigation of a subset of human polymorphisms by ligase detection reaction and universal array. Minerva Biotecnologica. 2002;14(3-4):259-63.
71. Zareparsi S, James DM, Kaye JA, Bird TD, Schellenberg GD, Payami H. HLA-A2 homozygosity but not heterozygosity is associated with Alzheimer disease. Neurology. 2002 Mar 26;58(6):973-5.
72. Vogelgesang S, Schroeder E, Walker LC, Pahnke J, Naubereit A, Walther R, et al. Activated microglia do not mediate the early deposition of Abeta in carriers of the apolipoprotein Eepsilon4 allele. Clin Neuropathol. 2002 May-Jun;21(3):99-106.
73. Masterman T, Zhang Z, Hellgren D, Salter H, Anvret M, Lilius L, et al. APOE genotypes and disease severity in multiple sclerosis. Mult Scler. 2002 Apr;8(2):98-103.

74. Billiard M, Ondze B. [Disorders of awakening. Second part: secondary disorders]. Rev Neurol (Paris). 2001 May;157(5):480-96.
75. Dandrea MR, Reiser PA, Gumula NA, Hertzog BM, Andrade-Gordon P. Application of triple immunohistochemistry to characterize amyloid plaque-associated inflammation in brains with Alzheimer's disease. Biotech Histochem. 2001 Mar;76(2):97-106.
76. Devaney JM, Pettit EL, Kaler SG, Vallone PM, Butler JM, Marino MA. Genotyping of two mutations in the HFE gene using single-base extension and high-performance liquid chromatography. Anal Chem. 2001 Feb 1;73(3):620-4.
77. Head E, Garzon-Rodriguez W, Johnson JK, Lott IT, Cotman CW, Glabe C. Oxidation of Abeta and plaque biogenesis in Alzheimer's disease and Down syndrome. Neurobiol Dis. 2001 Oct;8(5):792-806.
78. Le TV, Crook R, Hardy J, Dickson DW. Cotton wool plaques in non-familial late-onset Alzheimer disease. J Neuropathol Exp Neurol. 2001 Nov;60(11):1051-61.
79. McCusker SM, Curran MD, Dynan KB, McCullagh CD, Urquhart DD, Middleton D, et al. Association between polymorphism in regulatory region of gene encoding tumour necrosis factor alpha and risk of Alzheimer's disease and vascular dementia: a case-control study. Lancet. 2001 Feb 10;357(9254):436-9.
80. Perry RT, Collins JS, Harrell LE, Acton RT, Go RC. Investigation of association of 13 polymorphisms in eight genes in southeastern African American Alzheimer disease patients as compared to age-matched controls. Am J Med Genet. 2001 May 8;105(4):332-42.
81. Robinson Agramonte M, Dorta-Contreras AJ, Lorigados Pedre L. [Immune events in central nervous system of early and late onset Alzheimer's disease patients]. Rev Neurol. 2001 May 16-31;32(10):901-4.
82. Sampietro M, Caputo L, Casatta A, Meregalli M, Pellagatti A, Tagliabue J, et al. The hemochromatosis gene affects the age of onset of sporadic Alzheimer's disease. Neurobiol Aging. 2001 Jul-Aug;22(4):563-8.
83. Szpak GM, Lechowicz W, Lewandowska E, Bertrand E, Wierzba-Bobrowicz T, Gwiazda E, et al. Neurones and microglia in central nervous system immune response to degenerative processes. Part 1: Alzheimer's disease and Lewy body variant of Alzheimer's disease. Quantitative study. Folia Neuropathol. 2001;39(3):181-92.
84. Imamura K, Sawada M, Ozaki N, Naito H, Iwata N, Ishihara R, et al. Activation mechanism of brain microglia in patients with diffuse neurofibrillary tangles with calcification: a comparison with Alzheimer disease. Alzheimer Dis Assoc Disord. 2001 Jan-Mar;15(1):45-50.
85. Hallam DM, Capps NL, Travelstead AL, Brewer GJ, Maroun LE. Evidence for an interferon-related inflammatory reaction in the trisomy 16 mouse brain leading to caspase-1-mediated neuronal apoptosis. J Neuroimmunol. 2000 Oct 2;110(1-2):66-75.
86. Kusdra L, Rempel H, Yaffe K, Pulliam L. Elevation of CD69+ monocyte/macrophages in patients with Alzheimer's disease. Immunobiology. 2000 May;202(1):26-33.
87. McGeer PL, McGeer EG, Yasojima K. Alzheimer disease and neuroinflammation. J Neural Transm Suppl. 2000;59:53-7.
88. Shepherd CE, Thiel E, McCann H, Harding AJ, Halliday GM. Cortical inflammation in Alzheimer disease but not dementia with Lewy bodies. Arch Neurol. 2000 Jun;57(6):817-22.
89. Small GW. Investigations into geriatric psychiatry challenges: AAGP Senior Investigator Award 2000. Am J Geriatr Psychiatry. 2000 Fall;8(4):276-83.
90. Ende N. The Berashis cell: A review is it similar to the embryonic stem cell? Journal of Medicine. 2000;31(3-4):113-30.
91. Schindowski K, Leutner S, Muller WE, Eckert A. Age-related changes of apoptotic cell death in human lymphocytes. Neurobiol Aging. 2000 Sep-Oct;21(5):661-70.
92. Graeber MB. Genetics of neuroinflammation in Alzheimer disease. Neurogenetics. 1999 Sep;2(3):135-6.

93. Ballerini C, Nacmias B, Rombola G, Marcon G, Massacesi L, Sorbi S. HLA A2 allele is associated with age at onset of Alzheimer's disease. Ann Neurol. 1999 Mar;45(3):397-400.
94. Neill D, Curran MD, Middleton D, Mawhinney H, Edwardson JA, McKeith I, et al. Risk for Alzheimer's disease in older late-onset cases is associated with HLA-DRB1*03. Neurosci Lett. 1999 Nov 12;275(2):137-40.
95. Wang G, Achim CL, Hamilton RL, Wiley CA, Soontornniyomkij V. Tyramide signal amplification method in multiple-label immunofluorescence confocal microscopy. Methods. 1999 Aug;18(4):459-64.
96. Yermakova AV, Rollins J, Callahan LM, Rogers J, O'Banion MK. Cyclooxygenase-1 in human Alzheimer and control brain: quantitative analysis of expression by microglia and CA3 hippocampal neurons. J Neuropathol Exp Neurol. 1999 Nov;58(11):1135-46.
97. Lombardi VR, Garcia M, Rey L, Cacabelos R. Characterization of cytokine production, screening of lymphocyte subset patterns and in vitro apoptosis in healthy and Alzheimer's Disease (AD) individuals. J Neuroimmunol. 1999 Jun 1;97(1-2):163-71.
98. Bayer TA, Buslei R, Havas L, Falkai P. Evidence for activation of microglia in patients with psychiatric illnesses. Neurosci Lett. 1999 Aug 20;271(2):126-8.
99. Overmyer M, Helisalmi S, Soininen H, Laakso M, Riekkinen P, Sr., Alafuzoff I. Reactive microglia in aging and dementia: an immunohistochemical study of postmortem human brain tissue. Acta Neuropathol. 1999 Apr;97(4):383-92.
100. Aisen PS, Luddy A, Durner M, Reinhard JF, Jr., Pasinetti GM. HLA-DR4 influences glial activity in Alzheimer's disease hippocampus. J Neurol Sci. 1998 Nov 26;161(1):66-9.
101. Bowser R, Reilly S. Expression of FAC1 in activated microglia during Alzheimer's disease. Neurosci Lett. 1998 Sep 11;253(3):163-6.
102. Kobayashi K, Fukutani Y, Hayashi M, Miyazu K, Muramori F, Aoki T, et al. Non-familial olivopontocerebellar atrophy combined with late onset Alzheimer's disease: a clinico-pathological case report. J Neurol Sci. 1998 Jan 21;154(1):106-12.
103. Popovic M, Caballero-Bleda M, Puelles L, Popovic N. Importance of immunological and inflammatory processes in the pathogenesis and therapy of Alzheimer's disease. Int J Neurosci. 1998 Sep;95(3-4):203-36.
104. Styren SD, Kamboh MI, DeKosky ST. Expression of differential immune factors in temporal cortex and cerebellum: the role of alpha-1-antichymotrypsin, apolipoprotein E, and reactive glia in the progression of Alzheimer's disease. J Comp Neurol. 1998 Jul 13;396(4):511-20.
105. Combarros O, Escribano J, Sanchez-Velasco P, Leyva-Cobian F, Oterino A, Leno C, et al. Association of the HLA-A2 allele with an earlier age of onset of Alzheimer's disease. Acta Neurol Scand. 1998 Aug;98(2):140-1.
106. Curran M, Middleton D, Edwardson J, Perry R, McKeith I, Morris C, et al. HLA-DR antigens associated with major genetic risk for late-onset Alzheimer's disease. Neuroreport. 1997 Apr 14;8(6):1467-9.
107. Hollister RD, Xia M, McNamara MJ, Hyman BT. Neuronal expression of class II major histocompatibility complex (HLA-DR) in 2 cases of Pick disease. Arch Neurol. 1997 Mar;54(3):243-8.
108. Maat-Schieman MLC, Van Duinen SG, Rozemuller AJM, Haan J, Roos RAC. Association of vascular amyloid (beta) and cells of the mononuclear phagocyte system in hereditary cerebral hemorrhage with amyloidosis (Dutch) and Alzheimer disease. Journal of Neuropathology and Experimental Neurology. 1997;56(3):273-84.
109. McGeer EG, McGeer PL. The role of the immune system in neurodegenerative disorders. Mov Disord. 1997 Nov;12(6):855-8.
110. Yamada T, Tsuboi Y, Takahashi M. Interrelationship between beta-amyloid deposition and complement-activated oligodendroglia. Dement Geriatr Cogn Disord. 1997 Sep-Oct;8(5):267-72.
111. Pennisi E. Random samples. Science. 1997;277(5331):1441.

112. Payami H, Schellenberg GD, Zareparsi S, Kaye J, Sexton GJ, Head MA, et al. Evidence for association of HLA-A2 allele with onset age of Alzheimer's disease. Neurology. 1997 Aug;49(2):512-8.
113. Aisen PS. Inflammation and Alzheimer disease. Mol Chem Neuropathol. 1996 May-Aug;28(1-3):83-8.
114. Matsuo A, Walker DG, Terai K, McGeer PL. Expression of CD43 in human microglia and its downregulation in Alzheimer's disease. J Neuroimmunol. 1996 Dec;71(1-2):81-6.
115. Sandbrink R, Hartmann T, Masters CL, Beyreuther K. Genes contributing to Alzheimer's disease. Mol Psychiatry. 1996 Mar;1(1):27-40.
116. Tomimoto H, Akiguchi I, Suenaga T, Nishimura M, Wakita H, Nakamura S, et al. Alterations of the blood-brain barrier and glial cells in white-matter lesions in cerebrovascular and Alzheimer's disease patients. Stroke. 1996 Nov;27(11):2069-74.
117. Vidal R, Ghiso J, Wisniewski T, Frangione B. Alzheimer's presenilin 1 gene expression in platelets and megakaryocytes. Identification of a novel splice variant. FEBS Lett. 1996 Sep 9;393(1):19-23.
118. Garlepp MJ. Genetics of the idiopathic inflammatory myopathies. Curr Opin Rheumatol. 1996 Nov;8(6):514-20.
119. Tomimoto H, Akiguchi I, Wakita H, Kinoshita A, Ikemoto A, Nakamura S, et al. Glial expression of cytokines in the brains of cerebrovascular disease patients. Acta Neuropathol. 1996 Sep;92(3):281-7.
120. Shalit F, Sredni B, Brodie C, Kott E, Huberman M. T lymphocyte subpopulations and activation markers correlate with severity of Alzheimer's disease. Clin Immunol Immunopathol. 1995 Jun;75(3):246-50.
121. Uchihara T, Kondo H, Akiyama H, Ikeda K. Single-laser three-color immunolabeling of a histological section by laser scanning microscopy: application to senile plaque-related structures in post-mortem human brain tissue. J Histochem Cytochem. 1995 Jan;43(1):103-6.
122. Kimura H, Tooyama I, McGeer PL. Acidic FGF expression in the surroundings of senile plaques. Tohoku J Exp Med. 1994 Nov;174(3):279-93.
123. Liew SC, Penfold PL, Provis JM, Madigan MC, Billson FA. Modulation of MHC class II expression in the absence of lymphocytic infiltrates in Alzheimer's retinae. J Neuropathol Exp Neurol. 1994 Mar;53(2):150-7.
124. Maat-Schieman ML, Rozemuller AJ, van Duinen SG, Haan J, Eikelenboom P, Roos RA. Microglia in diffuse plaques in hereditary cerebral hemorrhage with amyloidosis (Dutch). An immunohistochemical study. J Neuropathol Exp Neurol. 1994 Sep;53(5):483-91.
125. Matsuse T, Namba Y, Ikeda K, Inoue S, Hosoi T, Ouchi Y, et al. Immunohistochemical and in situ hybridisation detection of adenovirus early region 1A (E1A) gene in the microglia of human brain tissue. J Clin Pathol. 1994 Mar;47(3):275-7.
126. Renkawek K, Voorter CE, Bosman GJ, van Workum FP, de Jong WW. Expression of alpha B-crystallin in Alzheimer's disease. Acta Neuropathol. 1994;87(2):155-60.
127. Frecker MF, Pryse-Phillips WE, Strong HR. Immunological associations in familial and non-familial Alzheimer patients and their families. Can J Neurol Sci. 1994 May;21(2):112-9.
128. Carpenter AF, Carpenter PW, Markesbery WR. Morphometric analysis of microglia in Alzheimer's disease. J Neuropathol Exp Neurol. 1993 Nov;52(6):601-8.
129. Barcikowska M, Liberski PP, Boellaard JW, Brown P, Gajdusek DC, Budka H. Microglia is a component of the prion protein amyloid plaque in the Gerstmann-Straussler-Scheinker syndrome. Acta Neuropathol. 1993;85(6):623-7.
130. Eisen A, Calne D. Amyotrophic lateral sclerosis, Parkinson's disease and Alzheimer's disease: phylogenetic disorders of the human neocortex sharing many characteristics. Can J Neurol Sci. 1992 Feb;19(1 Suppl):117-23.

131. Gardella JE, Gorgone GA, Newman P, Frangione B, Gorevic PD. Characterization of Alzheimer amyloid precursor protein transcripts in platelets and megakarocytes. Neurosci Lett. 1992 Apr 27;138(2):229-32.
132. Osterland CK, St Louis EA. Immune reactivity in aging-autologous mixed lymphocyte responses. Gerontology. 1992;38(6):301-7.
133. Perlmutter LS, Scott SA, Barron E, Chui HC. MHC class II-positive microglia in human brain: association with Alzheimer lesions. J Neurosci Res. 1992 Dec;33(4):549-58.
134. Bringuier AF, Seebold-Choqueux C, Moricard Y, Simmons DJ, Milhaud G, Labat ML. T-lymphocyte control of HLA-DR blood monocyte differentiation into neo-fibroblasts. Further evidence of pluripotential secreting functions of HLA-DR monocytes, involving not only collagen but also uromodulin, amyloid-beta peptide, alpha-fetoprotein and carcinoembryonic antigen. Biomed Pharmacother. 1992;46(2-3):91-108.
135. Akiyama H, Kawamata T, Dedhar S, McGeer PL. Immunohistochemical localization of vitronectin, its receptor and beta-3 integrin in Alzheimer brain tissue. J Neuroimmunol. 1991 Apr;32(1):19-28.
136. Frohman EM, Frohman TC, Gupta S, de Fougerolles A, van den Noort S. Expression of intercellular adhesion molecule 1 (ICAM-1) in Alzheimer's disease. J Neurol Sci. 1991 Nov;106(1):105-11.
137. Ikeda T, Yamamoto K, Takahashi K, Kaneyuki H, Yamada M. Interleukin-2 receptor in peripheral blood lymphocytes of Alzheimer's disease patients. Acta Psychiatr Scand. 1991 Sep;84(3):262-5.
138. Ikeda T, Yamamoto K, Takahashi K, Yamada M. Immune system-associated antigens on the surface of peripheral blood lymphocytes in patients with Alzheimer's disease. Acta Psychiatr Scand. 1991 Jun;83(6):444-8.
139. McGeer PL, McGeer EG, Kawamata T, Yamada T, Akiyama H. Reactions of the immune system in chronic degenerative neurological diseases. Can J Neurol Sci. 1991 Aug;18(3 Suppl):376-9.
140. Panegyres PK, Dawkins RL. Complement allotypes in familial and sporadic Alzheimer's disease. J Neurol. 1991 Sep;238(6):325-6.
141. Hassan NF, Campbell DE, Rifat S, Douglas SD. Isolation and characterization of human fetal brain-derived microglia in in vitro culture. Neuroscience. 1991;41(1):149-58.
142. Small GW, Matsuyama SS. HLA-A2 as a possible marker for early-onset Alzheimer disease in men. Neurobiol Aging. 1986 May-Jun;7(3):211-4.
143. Small GW, Ebeling SC, Matsuyama SS, Heyman A, Reisner EG, Renvoize EB, et al. Variable association of HLA-A2 in men with early-onset Alzheimer disease. Neurobiol Aging. 1991 Jul-Aug;12(4):375-7.
144. Akiyama H, McGeer PL. Brain microglia constitutively express beta-2 integrins. J Neuroimmunol. 1990 Nov;30(1):81-93.
145. Araga S, Kagimoto H, Funamoto K, Takahashi K. Lymphocyte proliferation and subpopulations in dementia of the Alzheimer type. Jpn J Med. 1990 Nov-Dec;29(6):572-5.
146. Grundke-Iqbal I, Fleming J, Tung YC, Lassmann H, Iqbal K, Joshi JG. Ferritin is a component of the neuritic (senile) plaque in Alzheimer dementia. Acta Neuropathol. 1990;81(2):105-10.
147. Mattiace LA, Davies P, Dickson DW. Detection of HLA-DR on microglia in the human brain is a function of both clinical and technical factors. Am J Pathol. 1990 May;136(5):1101-14.
148. Mattiace LA, Davies P, Yen SH, Dickson DW. Microglia in cerebellar plaques in Alzheimer's disease. Acta Neuropathol. 1990;80(5):493-8.
149. Rogers J, Mufson EJ. Demonstrating immune-related antigens in Alzheimer's disease brain tissue. Neurobiol Aging. 1990 Jul-Aug;11(4):477-9.

150. Styren SD, Civin WH, Rogers J. Molecular, cellular, and pathologic characterization of HLA-DR immunoreactivity in normal elderly and Alzheimer's disease brain. Exp Neurol. 1990 Oct;110(1):93-104.
151. Tooyama I, Kimura H, Akiyama H, McGeer PL. Reactive microglia express class I and class II major histocompatibility complex antigens in Alzheimer's disease. Brain Res. 1990 Jul 23;523(2):273-80.
152. Itagaki S, McGeer PL, Akiyama H, Zhu S, Selkoe D. Relationship of microglia and astrocytes to amyloid deposits of Alzheimer disease. J Neuroimmunol. 1989 Oct;24(3):173-82.
153. McGeer PL, Akiyama H, Itagaki S, McGeer EG. Immune system response in Alzheimer's disease. Can J Neurol Sci. 1989 Nov;16(4 Suppl):516-27.
154. Small GW, Matsuyama SS, Komanduri R, Spar JE, Fairbanks L. HLA antigens in depressed, demented, and nondemented elderly. J Geriatr Psychiatry Neurol. 1989 Apr-Jun;2(2):70-5.
155. Hofman FM, Hinton DR, Johnson K, Merrill JE. Tumor necrosis factor identified in multiple sclerosis brain. J Exp Med. 1989 Aug 1;170(2):607-12.
156. Itagaki S, McGeer PL, Akiyama H. Presence of T-cytotoxic suppressor and leucocyte common antigen positive cells in Alzheimer's disease brain tissue. Neurosci Lett. 1988 Sep 12;91(3):259-64.
157. Luber-Narod J, Rogers J. Immune system associated antigens expressed by cells of the human central nervous system. Neurosci Lett. 1988 Nov 22;94(1-2):17-22.
158. McGeer PL, Itagaki S, Boyes BE, McGeer EG. Reactive microglia are positive for HLA-DR in the substantia nigra of Parkinson's and Alzheimer's disease brains. Neurology. 1988 Aug;38(8):1285-91.
159. McGeer PL, Itagaki S, McGeer EG. Expression of the histocompatibility glycoprotein HLA-DR in neurological disease. Acta Neuropathol. 1988;76(6):550-7.
160. Rogers J, Luber-Narod J, Styren SD, Civin WH. Expression of immune system-associated antigens by cells of the human central nervous system: relationship to the pathology of Alzheimer's disease. Neurobiol Aging. 1988 Jul-Aug;9(4):339-49.
161. McGeer PL, Itagaki S, Tago H, McGeer EG. Occurrence of HLA-DR reactive microglia in Alzheimer's disease. Ann N Y Acad Sci. 1988;540:319-23.
162. Hulette CM, Walford RL. Immunological aspects of Alzheimer disease: a review. Alzheimer Dis Assoc Disord. 1987;1(2):72-82.
163. McGeer PL, Itagaki S, Tago H, McGeer EG. Reactive microglia in patients with senile dementia of the Alzheimer type are positive for the histocompatibility glycoprotein HLA-DR. Neurosci Lett. 1987 Aug 18;79(1-2):195-200.
164. Clemenceau S, Foncin JF, Muller JY, Halle L, Hauptmann G, Seger J, et al. [Absence of a connection between Alzheimer's disease and complement markers]. C R Acad Sci III. 1986;303(5):149-54.
165. Davies P. The genetics of Alzheimer's disease: a review and a discussion of the implications. Neurobiol Aging. 1986 Nov-Dec;7(6):459-66.
166. Hofman FM, von Hanwehr RI, Dinarello CA, Mizel SB, Hinton D, Merrill JE. Immunoregulatory molecules and IL 2 receptors identified in multiple sclerosis brain. J Immunol. 1986 May 1;136(9):3239-45.
167. Kay DW. The genetics of Alzheimer's disease. Br Med Bull. 1986 Jan;42(1):19-23.
168. Muller JY, Clemenceau S, Foncin JF. Absence of linkage between Alzheimer's disease and the HLA system. Comptes Rendus de l'Academie des Sciences - Serie III. 1986;303(4):105-8.
169. Hulette CM, Walford RL. HLA and complement studies in Alzheimer's disease. Bull Clin Neurosci. 1985;50:32-5.

170. Majsky A. HLA system and psychiatry. Analysis of results. Cesko-Slovenska Psychiatrie. 1985;81(3):171-5.
171. Nerl C, Mayeux R, O'Neill GJ. HLA-linked complement markers in Alzheimer's and Parkinson's disease: C4 variant (C4B2) a possible marker for senile dementia of the Alzheimer type. Neurology. 1984 Mar;34(3):310-4.
172. Renvoize EB, Hambling MH. Cytomegalovirus infection and Alzheimer's disease. Age Ageing. 1984 Jul;13(4):205-9.
173. The dementia syndrome. Lancet. 1983 Jan 22;1(8317):187.
174. Delasnerie-Laupretre N, Calot M, Ohayon E, Foucault C, Cambon-de-Mouzon A, Clanet M, et al. [Familial Alzheimer's disease: a study of HLA markers]. Biomed Pharmacother. 1983;37(4):186-8.
175. Eikelenboom P, Jonker C, Stam FC. [Do immunological factors play a role in the pathogenesis of Alzheimer's disease?]. Tijdschr Gerontol Geriatr. 1983 Oct;14(5):181-5.
176. Guard O, Dumas R, Besancenot JF, Guignier F, Laurin S. [The HLA system in Alzheimer-like dementias]. Presse Med. 1983 Apr 23;12(18):1171.
177. Weitkamp LR, Nee L, Keats B. Alzheimer disease: Evidence for susceptibility loci on chromosomes 6 and 14. American Journal of Human Genetics. 1983;35(3):443-53.
178. Good RA, Kapoor N, Reisner Y. Bone marrow transplantation - an expanding approach to treatment of many diseases. Cellular Immunology. 1983;82(1):36-54.
179. Harris R. Genetics of Alzheimer's disease. Br Med J (Clin Res Ed). 1982 Apr 10;284(6322):1065-6.
180. Nerl CW, Mayeux R, O'Neill GJ. Complement C4 allotypes in Alzheimer's disease. Lancet. 1982 Dec 11;2(8311):1343.
181. Cohen D, Eisdorfer C, Walford RL. Histocompatibility antigens (HLA) and patterns of cognitive loss in dementia of the Alzheimer type. Neurobiol Aging. 1981 Winter;2(4):277-80.
182. Goudsmit J, White BJ, Weitkamp LR, Keats BJ, Morrow CH, Gajdusek DC. Familial Alzheimer's disease in two kindreds of the same geographic and ethnic origin. A clinical and genetic study. J Neurol Sci. 1981 Jan;49(1):79-89.
183. Majsky A, Korinkova P, Fortynova J, Abrahamova J, Vojtechovsky M. Some critical remarks on the problem of HLA association with disease. Folia Haematol Int Mag Klin Morphol Blutforsch. 1981;108(3):386-91.
184. Wilcox CB, Caspary EA, Behan PO. Histocompatibility antigens in Alzheimer's disease. A preliminary study. Eur Neurol. 1981;20(1):25-8.
185. Renvoize EB, Hambling MH, Pepper MD, Rajah SM. Possible association of Alzheimer's disease with HLA-BW15 and cytomegalovirus infection. Lancet. 1979 Jun 9;1(8128):1238.
